# Rumen-protected choline: A significance effect on dairy cattle nutrition

**DOI:** 10.14202/vetworld.2016.837-841

**Published:** 2016-08-11

**Authors:** G. Jayaprakash, M. Sathiyabarathi, M. Arokia Robert, T. Tamilmani

**Affiliations:** 1 Department of Animal Nutrition, College of Veterinary and Animal Sciences, Kerala Veterinary and Animal Sciences University, Mannuthy, Thrissur - 680 651, Kerala, India; 2 Southern Regional Station, ICAR - National Dairy Research Institute, Adugodi, Bengaluru - 560 030, Karnataka, India; 3 Department of Poultry Science, Indian Veterinary Research Institute, ICAR, Izatnagar - 243 122, Uttar Pradesh, India

**Keywords:** choline, dairy cows, health, production, reproduction

## Abstract

Choline is a vitamin-like substance it has multi-function in animal production, reproduction, and health. The transition period is most crucial stage in lactation cycle of dairy cows due to its association with negative hormonal and energy balances. Unfortunately, unprotected choline easily degrades in the rumen; therefore, choline added to the diet in a rumen-protected form. The use of rumen-protected choline (RPC) is a preventive measurement for the fatty liver syndrome and ketosis; may improve milk production as well as milk composition and reproduction parameters. This review summarizes the effectiveness of RPC on animal production, health, and reproduction.

## Introduction

Choline [(CH_3_)3N+CH_2_CH_2_OH], also called as trimethyl ethanolamine, is a multi-function B-complex vitamin. It is synthesized endogenously, and it is difficult to identify a syndrome in ruminants because of its interrelationship with methionine, vitamin B12, and folic acid. Rumen-protected choline (RPC) acts on several mechanisms; first, it may serve to spare methyl groups. Second, it may serve as the remethylization of homocysteine via its metabolite betaine. Third, it may be spare animal metabolism. It is found in free form in biological tissues and component of lecithin, acetylcholine, plasmalogens, and sphingomyelins. During the transition period, cows may undergo a severe negative energy balance (NEBAL). NEBAL causes several metabolic disorders or disease in dairy cows. Immediate onset of lactation and increased milk production, animal require excess amount of energy. Recent research indicates feed additives, such as RPC, were observed decreases the health disturbances and increases the milk yield. Supplemental choline chloride extensively degraded (>80%) by rumen microbial population [1] and not much choline is available for absorption. Therefore, choline must be given in the rumen-protected form [2]. RPC usually supplied as choline chloride covered by a protective layer of the fatty acid matrix; rumen microbes cannot digest fatty acid layer of protected choline. However, the digestive enzyme breaks down the fatty acid in small intestine and choline is free for absorption. Choline has been observed to increase milk production [3-5]. Moreover, choline supplementation may improve the transport of lipids in the blood to reduce the risk of fatty liver and ketosis [6,7] and decreases the accumulation of non-esterified fatty acid (NEFA) concentration and increased glycogen content of liver [8]. RPC has a positive effect on decreasing hepatic triacylglycerides, and it may involve in increased fatty acid oxidation [6]. Supplementation of RPC to transition dairy cows can alleviate the health disorders, reproductive problems, the incidence of metabolic disorders [5]; an occurrence of ketosis and mastitis [9].

## NEFA as an Indicator of NEBAL

During calving, increased NEFA and beta hydroxyl butyric acid (BHBA) concentration in the blood indicate NEBAL and it leads to reduced milk yield, increased postpartum diseases, and decreased reproductive performance. The presence of NEFA in blood is a direct indicator for NEBAL. In high yielding dairy cows during the transition period, a rapid mobilization of fatty acids from the adipose tissue, resulting in high circulating concentrations of NEFA in the blood stream. Excessive mobilization of fatty acids from adipose tissue indicates that more energy is required than the dietary supplement. In ruminants limited capacities of hepatic fatty acid oxidation and export of very-low density lipoprotein (VLDL) [10]. Therefore, increase uptake of NEFA by the liver can result in the development of fatty liver (hepatic lipidosis caused by increased accumulation of triacylglycerol within liver parenchyma). Triglyceride fatty acids in chylomicrons and lipoprotein called as NEFA. In addition, excessive accumulation of triglyceride in the liver leads to alleviating the capacity of detoxifying ammonia to urea [11] which, in turn, may disturb gluconeogenic capacity from propionate, the predominant glucogenic precursor.

## Rumen Degradation of Dietary Choline

**Figure-1:**
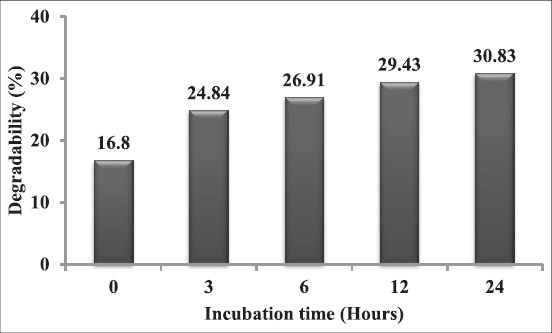
Percent degradability of rumen-protected choline by insacco method [14].

Study of unprotected choline in rumen not effective measure by digestibility studies because due to complete or partially degradation by rumen microbes before it even reaches the intestine [3] and difficult to estimate of endogenous choline synthesis in ruminants [12]. Synthetic choline chloride was more degradable than naturally occurring choline in the feed [2]. Sharma and Erdman [13] reported that the percentage of dietary choline and synthetic choline degradation in the rumen varies according to the type of feed (Table-1). Hence, supplementation of unprotected choline (conveniently as it is salt and choline chloride) ineffective way to increase the choline supply. Therefore, the rumen-protected form of choline has been developed to deliver choline with less degradation (Figure-1) to the small intestine for absorption [14]. Choline requirement is still unknown for dairy cows [12]. Rumen protected a form of choline increases the supply of choline to the small intestine with increasing milk yield and milk components or alleviating development of fatty liver syndrome [4,8,15]. To avoid ruminal degradation, researchers approached two types of experimental methods for delivering choline directly to the small intestine without ruminal degradation, *viz*., post-ruminal infusion, choline infused directly through the abomasum; this method was developed before the introduction of rumen protected technology [1,13] and supplementation of choline in the form of rumen protected, many studies have been conducted on efficacy of rumen protected technology (Table-2).

**Table-1:** Rumen degradability of dietary feed choline and synthetic choline [2].

Feeds	Rumen degradability (%)
Barley	79.4
Cottonseed meal	84.7
Fish meal	82.9
Soybean meal	83.8
Choline stearate	98.0
Choline chloride	98.6

**Table-2:** Description of RPC used in the different studies.

Study	Choline chloride % (wt/wt)	Rumen stability %	References
1	25	85^a^	[36]
2	25	20.4^b^	[37]
3	-	>85^c^	[3]
4	45	-	[7]
5	50	50^d^	[38]
6	27.11	72.89	[14]
7	18.8	-	[27]

a *In vitro* analysis,

b After 8 h *in situ* incubation in rumen cannulated adult ewes,

c After 48 h *in situ* incubation in rumen cannulated adult ewes,

d *In vitro* analysis. RPC: Rumen protected choline

### Impact of dry matter intake (DMI)

RPC supplements could not increase cow’s appetite [16]. Many researchers found that RPC did not affect DMI in early lactating dairy cows [16-19].

### Impact of choline on digestibility

Choline supplements increase to produce volatile fatty acid (VFA) acetate and rumen pH tends to increase digestibility coefficient of nutrients in rumen. Mohsen *et al*. [9] studied on the digestibility coefficient in Friesian cows supplemented with RPC, which increases DM, organic matter, crude protein, crude fiber, ether extract, and nitrogen free extract significantly. This might be because of RPC enhances the protozoal population in the rumen. In numerous studies, researchers found supplementation of RPC increases the DMI in dairy cows [20]. Contrary to the above findings, some researchers also found that the supplementation of RPC did not change the DMI [6,21]. Supplementation of RPC did not affect DMI before calving, but when supplemented after calving, it tended to increase DMI. It is unknown, the mechanism by which choline might influence DMI [22].

### Impact of choline on hematological parameter

RPC supplemented cows had significantly increased serum cholesterol [20] but reduced plasma cholesterol, triglycerides concentration [9], and glucose [8,23]. RPC had no significant effect on the levels of plasma concentration of NEFA, BHBA, and glucose. This might be because of no alteration in the adipose mobilization or BHBA production in the liver by choline supplementation [8,17,19,23]. Supplementation of RPC had no significant effect on the plasma glucose, total protein, albumin, globulin, and urea-N in different experimental groups [17,24].

### Impact of choline on milk yield

In an animal production system, the quantity and quality of milk production are the most important traits. Supplementation of RPC increases the milk production for the following reasons, *viz*., higher digestibility and increased total VFA concentration, decreased NH_3_-N, and prevention of metabolic disorders such as ketosis and fatty liver syndrome [9]. Increasing the intestinal supply of choline has improved milk production in lactating dairy cows approximately 7% over controls [25]. In numerous studies, researchers have been observed a tendency for higher milk yield (Table-3) with the supplementation of RPC [9,26] and fat corrected milk (FCM) [20,22]. In contrast, some researchers could not observe any positive effect on milk production and FCM by supplementation of RPC [24,27,28]. However, supplementation of RPC had no effect on milk yield for the following reasons, *viz*., the difference in study design such as body condition score [19], parity [18], other supplement diets [5], method of application [29], breed [17], and the quantity of supplement, length of the treatment period, and stage of lactation.

**Table-3:** Summary of studies on the impact of supplementation of RPC on milk production and composition on the lactating dairy cow.

Lactation stage	RPC (g/day)	DMI (kg/day)	Milk yield (kg/day)	Fat yield (g/day)	Protein yield (g/day)	References
21 days prepartum to 63 days postpartum	0	12.617.8	40.0	1593	1174	[8]
	45	11.918.7	43.3	1836	1314	
	60	12.818.3	39.9	1596	1206	
	75	12.518.8	41.0	1763	1262	
14 days prepartum to 30 days postpartum	0	11.319.4	28.5	880	868	[4]
	20	11.419.9	31.4	1056	966	
21 days prepartum to 21 days postpartum	0	12.014.8	29.6	1380	1050	[14]
	15	12.115.7	31.6	1460	1090	
21-90 days postpartum	0	27.9	27.9	840	730	[18]
	40	20.2	27.5	790	740	
4 weeks prepartum to 20 weeks postpartum	0	No	30.71	1071	1009	[5]
	60	reported	34.23	958	902	

RPC: Rumen protected choline, DMI: Dry matter intake

### Impact of choline on milk composition

Choline is essential lipotropic agent prevents and corrects excess fat deposition in the liver. Increase effect on milk protein by supplementation of choline due to elevated casein contents. Choline used facilitates phospholipid synthesis may lipid absorption and transport to the mammary gland, thereby favoring milk fat synthesis. Supplementation of RPC significantly increases the milk fat yield, milk protein, lactose, solids not fat (SNF), and total solids [8,9,21,24,27,30]. However, some researchers could not observe any significant effect on milk fat yield, milk protein, lactose, SNF, total solids, and milk urea nitrogen concentrations in RPC-supplemented cows compared with control [8,14,17,24,30,31]. On the other hand, supplementation RPC has not been positively associated with milk concentrations of lactose and other milk components [4,17,15].

### Impact of choline on health and body weight

In early lactation on dairy animals, the majority of fatty acids secreted by mammary gland and it can increase high animal weight decline, increase the incidence of fatty liver, and increase the chance of subclinical and clinical ketosis [6,4]. RPC supplements had weight gain in heifers [32], whereas RPC supplements found no effect on body weight [16,30]. During the transition period, accumulation fat in the liver and leads to fatty liver syndrome. Supplementation of RPC has been found to alleviate the range of hepatic fatty infiltration and increase VLDL transport from liver and prevents accumulation of fat in liver to avoid fatty liver syndrome [30]. RPC supplementation did not show any positive effect in reduction of fever, metritis, and displaced abomasum [33]. However, effectively reduces the incidence of clinical ketosis [34], mastitis, retained fetal membranes, and less morbidity [35]. RPC supplementation could not found any significant differences in body weightamong choline supplemented groups [8,20,27,30,33,35].

### Impact on reproductive performances

During the transition period, excess mobilization of adipose tissue to milk fat synthesis results in increased incidence of reproductive problems. Follicular development is less in during the period of NEBAL meanwhile follicles developed may be less chance of fertile. RPC did not influence the insemination and pregnancy loss [33]. Ardalan *et al*. [5] noticed a significant effect on service per conception and open days of dairy cows among the treatment groups (p<0.05), but there was no significant effect on days to first estrus and pregnant cows. Guretzky *et al*. [17] reported that supplemental RPC group had more twinning (p=0.07) compared to control group.

## Conclusion

During the transition period, RPC supplemented cows change plasma NEFA concentration, increase hepatic fat export, and this may effect decrease the risk for metabolic disorders and increase milk and milk composition.

## Authors’ Contributions

GJ conceptualized the concept of this review paper. GJ, MS, MA and TT drafted and edited the manuscript. All authors read and approved the final manuscript.
